# Hemolytic Uremic Syndrome Associated with *Escherichia coli* O8:H19 and Shiga Toxin 2f Gene

**DOI:** 10.3201/eid2101.140515

**Published:** 2015-01

**Authors:** Ingrid H.M. Friesema, Mandy G. Keijzer-Veen, Marja Koppejan, Henk S. Schipper, Arjanne J. van Griethuysen, Max E.O.C. Heck, Wilfrid van Pelt

**Affiliations:** National Institute for Public Health and the Environment, Bilthoven, the Netherlands (I.H.M. Friesema, M.E.O.C. Heck, W. van Pelt);; University Medical Center Utrecht, Utrecht, the Netherlands (M. G. Keijzer-Veen, H.S. Schipper);; Gelderse Vallei Hospital, Ede, the Netherlands (M. Koppejan, H.S. Schipper, A.J. van Griethuysen)

**Keywords:** Shiga toxin, Escherichia coli, bacteria, hemolytic uremic syndrome, renal, dialysis, zoonoses, virulence, Netherlands, Turkey, Germany

**To the Editor:** Gastroenteritis caused by Shiga toxin–producing *Escherichia coli* (STEC), associated with hemorrhagic colitis and hemolytic uremic syndrome (HUS), has been identified as a major health problem (*1*). Shiga toxin is essential for the development of HUS (*2*). Shiga toxin can be distinguished into Shiga toxin 1 (Stx1) and Shiga toxin 2 (Stx2). The *stx*_2f_ STEC variant is a distinct group within STEC (regarding virulence genes) and is known to cause relatively mild disease, although reports of human illness are scarce (*3*).

During autumn 2013, a healthy 9-year-old boy in the Netherlands experienced fever, vomiting, and bloody diarrhea which persisted for days; he was admitted to the pediatric ward of a local hospital because of clinical signs of HUS with renal insufficiency: serum creatinine level 439 μmol/L (reference range 31–68 μmol/L); blood urea nitrogen concentration 34.1 mmol/L (reference range 3.3–5.6 mmol/L); thrombocytopenia (46 platelets/nL; reference range 150–450/nL); and low haptoglobin level. Hemoglobin levels decreased within 48 hours from 7.4 mmol/L to 5.5 mmol/L (reference range 6.9–8.4 mmol/L). His blood pressure was 127/82 (99th percentile for age and height). Renal insufficiency worsened over time, evidenced by maximum urea levels of 57.3 mmol/L and maximum creatinine levels of 744 μmol/L. Vomiting increased, and feeding became difficult. The boy was transferred to an academic nephrology center, where he received erythrocyte and thrombocyte infusions, then peritoneal dialysis. He received 1 prophylactic dose of cefazolin during insertion of the dialysis catheter. After 2 days, he entered a polyuric phase of renal failure; renal function normalized within a few weeks, however. To improve proteinuria, physicians prescribed a 3-month course of angiotensin-converting enzyme inhibitors after discharge.

A fecal sample tested positive for STEC by PCR in a local laboratory. Five isolates were sent to the National Institute for Public Health and the Environment (RIVM) as part of the national STEC surveillance. By using PCR, 1 of the 5 tested positive for the *stx*_2f_ gene and the attaching and effacing gene (*eae*), and negative for the genes *stx*_1_, *stx*_2a-e_, H7, O157, and enterohemorrhagic *E. coli* hemolysin (*hly*). Serotyping identified O8:H19. The other 4 isolates tested negative for all of the above-mentioned genes and were not serotyped.

The family had stayed in a hotel in Turkey and returned to the Netherlands 5 days before onset of illness. The only reported contact with animals was with a parrot in the hotel. On return to the Netherlands, the boy had eaten filet américain, a sandwich spread made of raw beef. The day before disease onset, he attended a party where barbecue was served by a catering company.

Since 2007, besides this reported case, 8 cases of STEC O8 were registered within the STEC surveillance system in the Netherlands: O8:H– (4 cases), O8:H19 (2 cases), O8:H8 (1 case), and O8:H9 (1 case). All 8 isolates were *stx*_2a-e_-positive and *stx*_1_-, *stx*_2f_-, *eae-* and *hly-*negative. Disease associated with these cases was relatively mild. During 2007–2010, a total of 13,545 human STEC infections were reported in Europe: 20 were registered as STEC O8; HUS did not develop in these case-patients (*4*). HUS developed in 2 patients infected with STEC O8 (O8:H2, O8:H19) in Germany during 1996–2000 (*5*); these isolates and all other isolates from HUS and non-HUS case-patients in this period tested negative for *stx*_2f_. During 2008–2011, 87 *stx*_2f_ STEC infections were registered in the Netherlands (*3*). These infections were relatively mild; no HUS cases were registered. The virulence genes seen in the isolate of the described case, *stx*_2f_ and *eae*, but no *hly* or other toxin genes, were also seen in 97% of *stx*_2f_ STEC infections reported in the Netherlands (*3*). Besides being detected in humans, *stx*_2f_ STEC has only been detected in pigeons (*6*). 

The cause of the severity of disease in this *stx*_2f_ STEC case and the source of the infection could not be determined. The parrot in the hotel in Turkey could have been the source if birds are a reservoir of *stx*_2f_ STEC. Conversely, the uncooked beef and barbecue cannot be ruled out, because O8:H19 has been found in cattle, pigs, and sheep (*7*). This case shows that STEC subgroups known to cause relatively mild disease can occasionally cause severe disease and that surveillance based upon a small group of serotypes underestimates the number of severe STEC infections and increases the chance of missing emerging serotypes.
